# Comparison of the efficacy of sonic irrigation and conventional syringe irrigation in the removal of curcumin and triple antibiotic paste from root canals

**DOI:** 10.34172/joddd.2021.027

**Published:** 2021-08-25

**Authors:** Mehmet Adigüzel, Koray Yilmaz, İsmail İlker Pamukçu

**Affiliations:** ^1^Department of Endodontics, Faculty of Dentistry, Hatay Mustafa Kemal University, Hatay, Turkey

**Keywords:** Curcumin, Endodontics, Sonic activation, Triple antibiotic paste

## Abstract

**Background.** The present study aimed to compare the efficacy of sonic irrigation and conventional syringe irrigation (CSI) in terms of curcumin (CUR) and triple antibiotic paste (TAP) removal from a standardized groove artificially created in root canals.

**Methods.** The root canals of 72 anterior maxillary teeth were prepared using the Reciproc system to size R50. The teeth were split longitudinally, and a standardized groove was created in the apical region of one root half. The standardized grooves were filled with CUR or TAP with the exclusion of six teeth that served as the negative control group, and then the root halves were reassembled. The teeth were divided into two subgroups according to the irrigation protocols used: sonic activation with EndoActivator (EA) or CSI (n=15). After the removal of the medicament, the residual medicament was assessed under a stereomicroscope. Kruskal-Wallis and Mann-Whitney U tests were used for statistical analyses (*P* = 0.05).

**Results.** The EA sonic activation method was significantly more efficient in removing CUR medicament from the root canals. Considering the medicament types, more CUR than TAP was removed from the root canals using both CSI and the EA (sonic activation) system (*P* < 0.05).

**Conclusion.** As compared with CSI, the EA was not significantly more efficient in removing TAP, but it was significantly more effective than CSI in removing CUR.

## Introduction

Disinfection of the root canal system is the most significant factor affecting the success of root canal treatments.^1^ Many irrigation solutions and intracanal medicaments are used to disinfect root canals. In multi-session endodontic treatments, intracanal medicaments might be used to reduce intracanal bacterial counts and promote healing in the periapical zone. These medicaments should be completely removed from the root canal system before permanent root canal obturation. Failure to completely remove these medicaments might cause leakage of root canal filling and hamper the adhesion of root canal pastes to dentinal tubules.^2^

One material used as a medicament in the root canal system is triple antibiotic paste (TAP), which is frequently used in revascularization treatment. In previous studies, TAP exhibited a high level of antimicrobial activity, and it was effective in eliminating bacteria in infected root canals.^3-5^ However, TAP also has disadvantages, such as facilitating bacterial resistance to antibiotics, causing allergic reactions, and creating coronal discoloration due to its minocycline content.^6^ Curcumin (CUR), another intracanal medicament, is an organic polyphenolic compound obtained from the *Curcuma longa* L. (also known as turmeric) plant, with low molecular weight and hydrophobic nature. Previous studies have reported that CUR exhibited anti-inflammatory, antimicrobial, and antioxidant activities.^7,8^ CUR is considered an important intracanal medicament for root canal infections resistant to treatment. In studies using CUR as an intracanal medicament or irrigation solution, it exhibited superior antimicrobial efficacy, and CUR combined with piperine completely suppressed the formation of osteoclasts. Moreover, antioxidant, antiproliferative, and anticarcinogenic effects of CUR were enhanced light irradiation at an appropriate wavelength.^9-12^

In endodontics, activation systems are used to increase the efficacy of irrigation agents and effectively remove intracanal medicaments. EndoActivator (EA, Dentsply, Tulsa Dental, Tulsa, OK) is a sonic irrigation activation system. The EA portable handpiece system, which is equipped with noncutting polymer tips, agitates the irrigation solution in the root canal via sonic action. Previous studies reported that the irrigation action of the EA device was related to hydrodynamic activity arising from its pecking motion in the root canal.^13,14^

No studies are available on the removal of CUR medicament from root canals. In this study, the performance of the EA was compared with that of CSI to remove CUR and TAP from a standardized groove artificially created in root canals. The null hypothesis was that the EA system would result in no significant difference in the amount of CUR or TAP remaining in the root canals.

## Methods

### Root canal preparation

Seventy-two upper central incisor teeth with a single root and straight root canal were randomly selected from teeth with similar morphologies and sizes. In buccolingual and mesiodistal directions, preoperative periapical radiographs were taken, and teeth with cracks, fractures, caries, or resorption on the root surface were excluded. In addition, teeth with previous root canal treatments or fillings were excluded.

Soft tissues and hard tissue formations were mechanically removed from the root surfaces using a periodontal curette. The teeth were then stored in pure filtered water up to experimental procedures. The length of each tooth was standardized to 14 mm under water cooling by cutting at the cementoenamel junction using a 943DC diamond saw (Meisinger, Germany). The endodontic access cavities were prepared under water cooling using a high-speed diamond bur. A #15 K-file was placed into each canal until it was visible at the apical foramen, and apical patency was confirmed. The working length was determined 1 mm shorter than this measurement. An X-Smart Plus (Dentply Maillefer) endodontic motor and Reciproc R25, R40, and R50 (VDW, Munich, Germany) files were used to prepare the root canals. During root canal preparation, each root canal was irrigated after every three pecking motions using a 30-gauge irrigation needle (NaviTip; Ultradent Products, South Jordan, UT) and 2 mL of 5.25% sodium hypochlorite (NaOCl) solution. Equal amounts (10 mL) of NaOCl were used for each tooth until final irrigation. After completing the root canal preparation, the root canals were irrigated with 5 mL of 17% ethylenediaminetetraacetic acid (EDTA) for 60 seconds and 5 mL of 5.25% NaOCl for 60 seconds. The root canals were then rinsed with 10 mL of distilled water and dried using paper points.

The samples were fixed in silicon impression material and placed in Eppendorf tubes (Coltene Whaledent). After removing the impression material, the teeth were halved in a buccolingual direction using a diamond disc and a small chisel. A groove, 3 mm length, 0.2 mm in width, and 0.5 mm in depth, was then artificially created on each half at a distance of 2–5 mm from the apical region on the root canal wall. A toothbrush was used to remove the residue from the root surfaces and grooves. In the final irrigation, 5 mL of 17% EDTA was used for 60 seconds, followed by 5 mL of 5.25% NaOCl for 60 seconds. The root canals and artificially created grooves were then dried using paper points and an air current.

Subsequently, the teeth were divided into the following four groups (n = 15 in each group) and irrigated using either the EA (Dentsply) system or conventional syringe irrigation (CSI): TAP+CSI, TAP+EA, CUR+CSI, and CUR+EA. Six randomly selected specimens from the TAP (n = 3) and CUR (n = 3) groups served as negative controls. These specimens were not subjected to any other procedures. The grooves on the remaining teeth were filled with TAP, and the grooves on the other half were filled with CUR.

### Preparation of TAP

Equal amounts of metronidazole (Eczacibaşi, Istanbul, Turkey), doxycycline (Actavis, Istanbul, Turkey), and ciprofloxacin (Biofarma, Istanbul, Turkey) were mixed with distilled water in a 1:1 powder-to-liquid ratio.

### Preparation of CUR

CUR was prepared by mixing the medicament with distilled water (2.5 mg/mL). When preparing CUR, photoactivation using an LED device was used to increase its antimicrobial efficacy, as recommended for medicaments for clinical use.^10^ During photoactivation, the LED device was held at 90º at a specific distance. The LED light-curing unit (Bluephase LED; Ivoclar-Vivadent, Schaan, Lichtenstein) was operated at a wavelength of 385–515 nm. The radiation density was 1.200 mW/cm^2^. Photo-activation was performed for 4 minutes, with an interval of 30 seconds between each application.

The halved root surfaces were then reassembled using a small amount of cyanoacrylate glue. The endodontic access cavities were closed using a cotton pellet and a temporary filling material. The teeth were then placed in Eppendorf tubes and stored for two weeks at 100% moisture and 37°C temperature. After two weeks, six specimens from the TAP and CUR groups were randomly selected and used as positive controls. These specimens were subjected to no medicament removal procedure.

### Irrigation Protocols

#### TAP+CSI and CUR+CSI Treatment Groups

In removing the TAP or CUR medicament from the artificial grooves, a 30-gauge NaviTip syringe (Ultradent, USA) was used to irrigate each canal with 5 mL of 5.25% NaOCl and 5 mL of EDTA, with the syringe placed 1 mm shorter than the working length. The duration of the intracanal application was 1 minute for each irrigation solution. The total duration of the NaOCI and EDTA application was 2 minutes in all the groups. Finally, to prevent further irrigation effects, the specimens were irrigated using 5 mL of distilled water. For the final irrigation, the total volume of the irrigation solution was 15 mL in all the groups. The root canals were then dried using paper points.

#### TAP+EA and CUR+EA Treatment Groups

In total, 5 mL of 5.25% NaOCL was applied to the root canal space and pulpal chamber using a conventional syringe. The EA tip (#25/0.04) was then placed in the root canal at 2 mm shorter than the working length and used with vertical stroke motions at a speed of 10.000 cycles/minute. The root canals were then irrigated with 5 mL of 17% EDTA, and the EA was activated for 1 minute. As in the TAP+CSI and CUR+CSI groups, the specimens were then rinsed with 5 mL of distilled water to prevent a further irrigation effect. The root canals were then dried using paper points.

### Image Evaluation

The roots were separated to assess the residual amounts of medicaments in the TAP+CSI, CUR+CSI, TAP+EA, and CUR+EA groups. Images were obtained at ×20 magnification using a stereomicroscope (Olympus BX43; Olympus Co., Tokyo, Japan) connected to a digital camera, and the images were then transferred to a computer. Two endodontists (MA and KY) blinded to the groups assessed the amount of TAP and CUR remaining in the grooves according to the 4-point scoring system of van der Sluis et al^15^ :

0 = an empty cavity

1 = less than half the cavity is full of the tested medicament

2 = more than half the cavity is full of the tested medicament

**Figure 1:**
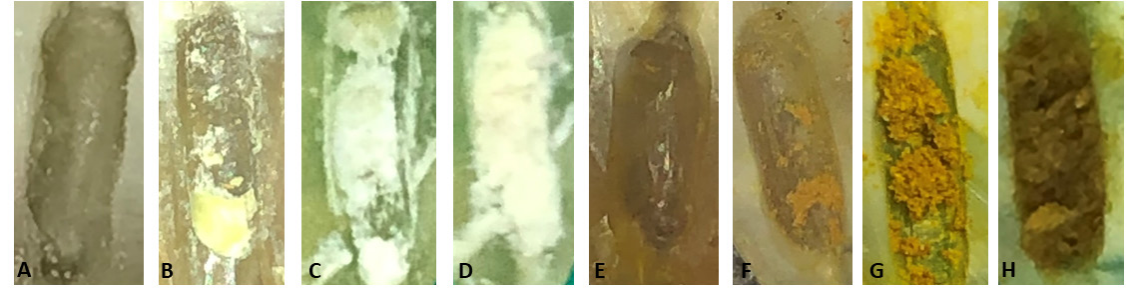
Representative images of scores for TAP ([A] score 0, [B] score 1, [C] score 2, and [D] score 3) and CUR ([E] score 0, [F] score 1, [G] score 2, and [H] score 3).

3 = the cavity is full of the tested medicament (Figure 1).

### Statistical analysis

The Shapiro–Wilk test was used to assess the normality of the data distribution. The data were statistically analyzed with Kruskal–Wallis and Mann–Whitney U tests, using SPSS 21 (IBM Inc., Chicago, IL, USA).

## Results

As determined using the kappa test, the inter-researcher agreement was 94.8%. Table 1 presents the scores for the medicament residue in the artificial grooves in each experimental group. All the specimens in the positive control group (root canals filled with CUR or TAP) had a score of 3, and all the specimens in the negative control group (root canals not filled with CUR or TAP) had a score of 0. The EA sonic activation method was significantly more efficient in removing CUR medicament from the root canal than that of CSI, with no significant effect on TAP medicament removal (*P* < 0.05). Considering the medicament types, more CUR than TAP was removed from the root canal using both CSI and the EA (sonic activation) system (*P* < 0.05).

**Table 1:** Score frequencies for the amounts of medicament removed in the experimental groups

**Group**	**n**	**Scores**			
		0	1	2	3
Negative control	6	6 (100%)	0 (0%)	0 (0%)	0 (0%)
Positive control	6	0 (0%)	0 (0%)	0 (0%)	6 (100%)
TAP+CSI	15	0 (0%)	4 (26.7%)	8 (53.3%)	3 (20.0%)
TAP+EA	15	3 (20.0%)	5 (33.3%)	6 (40.0%)	1 (6.7%)
CUR+CSI	15	5 (33.3%)	5 (33.3%)	5 (33.3%)	0 (0%)
CUR+EA	15	9 (60.0%)	6 (40.0%)	0 (0%)	0 (0%)

## Discussion

Complete removal of root canal medicament is crucial for 3D obturation of the root canal.^16^ Various methods, such as stereomicroscopes, scanning electron microscopes, and micro-CT, have been used to assess medicaments remaining on root canal walls.^17,18^ In the present study, we assessed the amount of medicaments (TAP and CUR) remaining, under a stereomicroscope at ×20 magnification using a scoring system similar to that used in previous studies.^3,19^

The complex anatomy of a natural root canal system cannot be simulated using standard groove models.^18^ However, using standardized dimensions, the groove model allows an assessment with high intraexaminer reproducibility and good interexaminer agreement.^20^ In the present study, we established a groove model by creating a standardized artificial groove in the apical region of root canals.

Disinfection of the apical third using a large volume of irrigation solution is difficult due to its complex anatomy. The removal of intracanal medicaments from the apical third is also more difficult than removing medicaments from the coronal and middle thirds. The use of NaOCl, followed by EDTA, is recommended to ensure sufficient disinfection in the root canal system, including the apical third.^21^ The efficacy of intracanal medicament removal can be increased by using different activation systems.^21^ In this study, the efficacy of the EA system was compared with that of CSI in CUR and TAP medicament removal. According to the results, the amount of medicaments (CUR and TAP) removed differed using the EA (Dentsply) system compared to that using CSI. Thus, the null hypothesis was rejected.

In this study, more CUR than TAP was removed from the root canals, irrespective of which irrigation method was used (i.e., the EA system or CSI). No studies have investigated the efficacy of irrigation systems in removing CUR medicament from the root canal system. Various studies have examined the antimicrobial aspects of CUR medicament. Devaraj et al^22^ investigated the effects of five intracanal medicaments (photo-activated CUR, TAP, double antibiotic paste [DAP], calcium hydroxide, and chlorhexidine) on *Enterococcus faecalis* biofilms in vitro. According to their results, photo-activated CUR exhibited superior antibiofilm and antibacterial activities against *E. faecalis* compared with those of TAP, with no significant difference between the groups. Moreover, photo-activated CUR and TAP caused significant degradation of biofilm structure compared with chlorhexidine and calcium hydroxide. Sotomil et al^23^ tested the antimicrobial activity of CUR and its potential use in root canal disinfection. According to their results, CUR was a potential alternative to TAP in controlling infections.

In the present study, significantly less TAP than CUR was removed from the root canals using CSI and sonic activation. Many studies have investigated the removal of TAP medicament from root canals. Using CSI, the EA system, and photon-initiated photoacoustic streaming, Arslan et al^24^ analyzed the removal efficacy of TAP and DAP medicaments from artificially established grooves. They reported no significant difference between the amounts of medicament removed, irrespective of the irrigation system applied. Berkhoff et al^25^ used a quantitative method to measure the amounts of TAP and calcium hydroxide residues within the root canal system and reported that radiolabeled calcium hydroxide was removed from the root canal systems four times more efficiently than radiolabeled TAP. They also reported that the removal of radiolabeled TAP from the root canal system was difficult and that none of the activation systems used (i.e., EA, EndoVac, a Max-i-Probe needle, or passive ultrasonic irrigation) could efficiently remove TAP from root canal systems due to the penetration and adhesion of TAP to the dentin. In the present study, TAP was more difficult than CUR to remove from the root canals using both CSI and sonic irrigation. The high diffusion and retention characteristics of TAP are due to the active substances and auxiliary materials used in the preparation of the medicament. The composition of TAP might explain why it is more resistant than CUR to removal. In contrast to TAP, CUR has more organic and additive-free content. Due to its composition, CUR medicament is more susceptible than TAP to the organic tissue dissolution properties of NaOCl.

In this study, the EA system was efficient in removing CUR but not efficient in removing TAP. Some previous studies reported that the EA system increased the efficacy of root canal medicament removal,^26,27^ whereas others reported that it did not.^28^ The divergent results might be related to methodological variables in studies. Sariyilmaz et al^29^ investigated the DAP and TAP removal efficacies of various irrigation protocols (passive ultrasonic irrigation, XP-endo finisher, CSI, and EA) and reported similar efficiency values for CSI and EA for both TAP and DAP. Thakur et al^30^ compared the efficacies of a canal brushing method, a sonic method (EA), and master apical file in removing TAP and reported that the canal brush and EA resulted in no significant difference in TAP removal from the apical third of the canal compared to the master apical file. Topçuoğlu et al^28^ compared the efficacies of various irrigation methods in removing calcium hydroxide from an artificially created internal root resorption cavity and found no significant difference between CSI and EA groups, consistent with the present study.

Akman et al^27^ compared the efficiencies of different irrigation activation protocols and the CSI method to remove modified TAP from root canal walls. They reported that the EA system significantly increased TAP removal from root canals compared with that of CSI. Similarly, Can et al^26^ compared the performance of EA, EndoVac, CanalBrush, and CSI systems in the removal of TAP, concluding that the EA significantly improved TAP removal from the root canal as compared with that of CSI. The fact that TAP removal using the EA was efficient in previous studies but not efficient in the present study can be explained by the use of complete root canals in these studies rather than standard apical grooves, as well as the methods used.

## Conclusion

As compared with CSI, the EA was not significantly more efficient in removing TAP, but it was significantly more effective than CSI in removing CUR. Using both irrigation methods, more CUR than TAP medicament was removed from the root canals. None of the irrigation protocols tested completely removed TAP or CUR from the root canals.

## Authors’ Contributions

MA, KY, and IIP were responsible for the concept. MA and IIP carried out the formal analysis. MA and KY were responsible for the interpretation of data. MA, KY, and IIP were responsible for the investigation. MA, KY, and IIP designed the methodology. MA, KY, and IIP were responsible for the supervision. MA, KY, and IIP prepared the original draft. MA and KY reviewed and edited the final draft.

## Funding

No funding.

## Competing Interests

The authors declare no conflict of interests.

## Ethics Approval

This study was approved by the Ethics Committee of Hatay Mustafa Kemal University (No.16/01/2020-04).
